# Alu elements contain many binding sites for transcription factors and may play a role in regulation of developmental processes

**DOI:** 10.1186/1471-2164-7-133

**Published:** 2006-06-01

**Authors:** Paz Polak, Eytan Domany

**Affiliations:** 1 Department of Physics of Complex Systems, Weizmann Institute of Science, Rehovot, 76100, Israel

## Abstract

**Background:**

The human genome contains over one million Alu repeat elements whose distribution is not uniform. While metabolism-related genes were shown to be enriched with Alu, in structural genes Alu elements are under-represented. Such observations led researchers to suggest that Alu elements were involved in gene regulation and were selected to be present in some genes and absent from others. This hypothesis is gaining strength due to findings that indicate involvement of Alu elements in a variety of functions; for example, Alu sequences were found to contain several functional transcription factor (TF) binding sites (BSs). We performed a search for new putative BSs on Alu elements, using a database of Position Specific Score Matrices (PSSMs). We searched consensus Alu sequences as well as specific Alu elements that appear on the 5 Kbp regions upstream to the transcription start site (TSS) of about 14000 genes.

**Results:**

We found that the upstream regions of the TSS are enriched with Alu elements, and the Alu consensus sequences contain dozens of putative BSs for TFs. Hence several TFs have Alu-associated BSs upstream of the TSS of many genes. For several TFs most of the putative BSs reside on Alu; a few of these were previously found and their association with Alu was also reported. In four cases the fact that the identified BSs resided on Alu went unnoticed, and we report this association for the first time. We found dozens of new putative BSs. Interestingly, many of the corresponding TFs are associated with early markers of development, even though the upstream regions of development-related genes are Alu-poor, compared with translational and protein biosynthesis related genes, which are Alu-rich. Finally, we found a correlation between the mouse B1 and human Alu densities within the corresponding upstream regions of orthologous genes.

**Conclusion:**

We propose that evolution used transposable elements to insert TF binding motifs into promoter regions. We observed enrichment of biosynthesis genes with Alu-associated BSs of developmental TFs. Since development and cell proliferation (of which biosynthesis is an essential component) were proposed to be opposing processes, these TFs possibly play inhibitory roles, suppressing proliferation during differentiation.

## Background

Over 90% of the human and other vertebrate genomes don't have any known functional role [1,2], and as such were considered until recently as "Junk DNA" or genomic "dark matter". A large fraction of the "non-functional" DNA originated from mobile elements [1] such as Alu, which comprise about 10% of the nucleotides of the human genome [1] with over one million inserted copies. This abundance is somewhat of a surprise, since Alu is a non-autonomous retroelement, i.e. it doesn't encode proteins that assist its mobilization, and therefore it needs to rely on the cell's machinery for its duplication in the genome [3]. Currently Alu is believed to be a parasite of the mobilization machinery of long interspersed elements (LINE) [4].

A typical Alu is a dimer, comprised of a central A-rich region which is flanked by two similar sequence elements of about 130 bp (left and right arms). During evolution the Alu elements have spread in the human genome in several bursts at different times, which allow their classification into several families and subfamilies, on the basis of their insertions, deletions and mutations. The major families are: old AluJ, whose members are AluJb and AluJo (both spread in the genome 80–100 mya); the middle, AluS family, which includes AluSx, AluSg, AluSp AluSc and AluSq (35 –50 mya), and the youngest Alu family, AluY (25 mya) [5,6].

One of the biggest mysteries associated with Alu concerns the non-random manner in which these elements are distributed throughout the human genome [1,7]. The distribution of Alu was found to be highly correlated with local GC content and with the density of genes and with intron density [8]. Furthermore, the density of Alu is higher in intragenic (12.5% of the nucleotides) than in intergenic regions (9.6% of the nucleotides) [8], which is surprising if indeed Alu elements are non-functional bits of DNA. In addition, Alu elements are negatively selected in imprinted regions of primate genomes [9]. Transposable elements are underrepresented in the region surrounding the TSS of genes [10]. Another analysis revealed that almost 20% of the genes contain transposable elements in the 3' UTR [11]. Significant differences were observed also when Alu density was compared among full length gene sequences of different biological process classes (this study [12] was limited to chromosomes 21 and 22). Genes that were related to metabolism, transport and signalling were mostly Alu rich, whereas genes that had poor Alu content belonged mostly to structural proteins and to information processing and storage pathways [12]. It was also shown recently that housekeeping genes contain more Alu than tissue specific genes [13].

This non-random of distribution of Alu, in particular the high concentration near and within genes, might be the result of positive feedback; selective pressure drives the organism to use these elements for some new functions, and the functions acquired by Alu generate advantage for phenotypes that have high Alu concentration near genes. Indeed, the possible functional roles of Alu in genomic organization and gene expression has received increasing attention during the last two decades (see [14,15] for reviews). One of the more fascinating possible consequences of integration of mobile elements near genes is acquisition of a transcriptional regulatory role by the Alu element, as a carrier of different TF binding sites. The Alu element harbours BSs of several transcription factors that were shown to bind to Alu; most of these TFs were nuclear factors, hormones, calcium nuclear factors, as well as other TFs [16-22]. The idea of Alu acting as a carrier of cis regulatory elements was suggested by Britten [23] and by Oei at el. [20] who found YY1 and SP1 BSs on young Alu family members. These experimental results imply that Alu elements have influence on the regulation of expression levels of many genes that have Alu in their promoter regions; however, such a wide ranged regulatory impact has not yet been tested experimentally.

The main aim of our study was to find new putative BSs that reside on Alu. Very recently, several groups have performed similar analysis for all transposable element classes; however, the set of PSSMs studied [24,25] contained about half of what is presented here. In addition, our analysis is complemented by an extensive literature search of past experimental work on the Alu-associated binding sites that we found, as well as the biological functions of the corresponding TFs and target genes. We started by searching the Alu *consensus sequences *and found new, previously unnoticed BSs, many of which turned out to be related to developmental processes.

The regulatory role played by Alu for genes that are related to differentiation or development was already established for at least six genes [26]. In these cases Alu was shown to act as enhancer or silencer involved in regulating the expression of six developmental genes. For one of these, the T-cell marker gene *CD*8α, the silencing mechanism was shown to act via binding of *LYF-1*, *bHLH *and *GATA3 *to the Alu situated in the last intron of *CD*8α [27,28]. These experimental results demonstrated the regulatory impact of Alu on developmental genes and during developmental stages. To explore further and enhance the possible regulatory roles of Alu we focused on the genomic region most likely to have a regulatory function – the upstream regions of genes. We scanned these regions and searched for BSs on the *particular Alu sequences *that were identified. Our findings show for the first time that TFs that are known to regulate the developmental processes may bind to Alu elements that are incorporated in the *promoter regions *of genes that need to be activated or suppressed during development (such binding has already been demonstrated for Gfi-1, PITX2 and Nkx2.5 – see below).

## Results and discussion

### The Alu consensus sequences are enriched with Binding Motifs

We focused on the eight major Alu subfamilies AluJo, Jb, Sx, Sz, Sc, Sq, Sp and Y [3], whose sequences were downloaded from RepBase [29]. We refer to the ensemble of sequences that bind a TF as it's Binding Motif (BM). In order to find BMs of a TF, we used its Position Specific Score Matrix (PSSM); 410 PSSMs were downloaded from two databases [30,31]. For each PSSM we set two threshold scores, T5 and T1, selected so that the probability to find a target BM with score higher than T5 in a suitably chosen random sequence (see Methods) is less than 0.05 (0.01 for T1). Furthermore, for each BM we identified Tmax, the maximal score observed on all the above listed Alu subfamilies' consensus sequences. For 66 BMs Tmax was higher than T5 and for 25 TFs – higher than T1 (see Table  1). A good measure for the statistical significance of enrichment of Alu with BMs is the number of BMs whose score exceeds T5; for example, we found on AluSx 35 BMs with scores higher than their respective T5. The probability to find this many BMs in a random model (i.e. p-value) is 0.023 (see Methods).

In order to control the problem of multiple testing we performed a false discovery rate (FDR) analysis at 20% FDR[32]. The 14 BMs that passed the FDR test with Q = 0.20 in AluSx are listed in Table 1, where we also compare the IUPAC consensus sequences of these BMs [33] with the target sequence that had the highest score in all the major Alu subfamilies. Most sequences with scores higher than T1 differ at not more than two nucleotides from the IUPAC consensus (see Table 1 and Additional data file 1), and there are several PSSMs, such as PITX2_Q2, LYF-1.01, PAX4.01, SIX3.01, DELTAEF.01 and NKX25.01 (Table 1, and Additional data file 1), whose IUPAC consensus sequences agree with the highest-scoring target sites on at least one of the Alu consensus sequences.

### BMs tend to cluster together on specific parts of Alu consensus sequences, that also tend to be more conserved during evolution

We checked the BM's locations along the consensus sequences on both strands of the eight Alu subfamilies. In general, most of the putative BSs were found on the sense strand. It is interesting to note that Alu elements show a 55% to 45% bias towards antisense orientation [34] relative to nearby genes. It would be of considerable interest to check experimentally whether this orientational difference is reflected in functional roles of the TFs. The target sites that passed T5 cover, on average, 50% of the consensus sequence of each subfamily. On the other hand, the total length of all the binding motifs found on one Alu consensus sequence exceeds, on the average, 420 nucleotides (150% of the Alu's length). This redundancy in putative target sites is caused by the fact that several PSSMs have very similar structure, giving top scores to the same DNA sequence.

The short sequences GGTAATCCC on the 5' to 3' strand and its complementary sequence GGGATTACC serve as a putative BS of five TFs, four of which are homeodomain proteins (Table 1 and Additional data file 1, TFs and locations marked by *). This sequence is part of the consensus of all eight Alu subfamilies, and in the five middle AluS subfamilies the BS appears on both the left and right monomers. Moreover, this is precisely the sequence identified by Britten [23] as one of the most conserved subsequences on Alu, which was seen as a surprise since it did not have any role in retrotransportation or for any other function. Later on, it was found that in the germ line the Specific Alu Binding Protein (SABP) can bind to this sequence and prevent methylation [35], but since the importance of this function of is still not understood, and hence the high level of conservation is still a mystery. Another region is a putative harbour for several BMs is the middle poly A- stretch (that bridges the two Alu monomers). We found this sequence to be a putative target site for many binding motifs such as all MEF2 family members, HNF1.03, OC.2 and PAX4 (marked by ** in Table 1 and Additional data file 1). These putative target sites were also found to accumulate less mutations than the expected rate [23]. More recently both the central poly A stretch and the subsequence mentioned above [36-38] were found to be associated with A-to-I editing.

### Existing evidence for TF binding to Alu (on DNA in solution)

Having identified BSs of various TFs on Alu, we searched for existing experimental evidence for binding and regulation associated with these motifs. Indeed, several TFs that are known to bind to motifs that passed our thresholds have already been shown to bind to DNA, and the fact that the binding sequences reside on Alu has been noticed (denoted by * on Table 2). We show in Table 2 four additional BMs that we found and report here for the first time; their corresponding TFs were shown to bind to the target sites identified by us, but the fact that they reside on Alu has not been previously noted. These TFs (bold in Table 2) are PITX2, Nkx2.5 Gfi-1 and LUN1. The most striking example was LUN-1; Chu et al. [39] observed (by EMSA) that it binds to 55 DNA sequences, 27 of which are palindromic; we discovered that all of these 27 reside on Alu. The situation is different for Gfi-1; there the PSSM [30] is based on 21 target sites, only five of which are part of Alu (Table 3). As to PITX2, it's PSSM [31] was based on target sites on the promoter of *PLOD1*, all of which reside on Alu subsequences [40]. Finally, the heart development marker Nkx2.*5 *is the only one whose PSSM was calculated using only off-Alu target sites – nevertheless, the target site found by us, that resides on Alu, is the one with the highest possible score.

### Evidence (in live cells) for regulation by TFs bound to Alu

The regulatory role of single – gene BSs on Alu was demonstrated for several TFs by a transfection assay for hormone ligands [16,22], for YY1 [20], and for Estrogen Receptors (ER) [18]. We found experimental evidence indicating that Gfi-1, PITX2 and Nkx2.5 regulate a reporter gene's expression [40-42] via BSs that reside on Alu; where the PITX2 function was validated in two different experiments [40,42]. In addition, Alu was essential for regulation of *PLOD1 *by PITX2, shown by using two constructs of *PLOD1 *promoter: one with several Alu elements and one without any Alu. These researchers [40] found a 3-fold change in the induction of a reporter gene with constructs that contained several Alu elements with respect to the expression level of the construct from which Alu elements were excluded. The same procedure of comparing promoters with and without Alu was done again on *PLOD1 *[42], but this time they checked PITX2 together with Nkx2.5 in order to prove the synergic effect on *PLOD1 *expression, and again a big difference between the expression levels of the reporter gene was found.

**ChIP analysis **was done on 21 genes' promoters that contain Gfi-1 putative target sites; five of these promoters contained suspected BSs on Alu (table 3). However, only one of these five genes (*JAK3*) had its Gfi-1 BS exclusively on Alu [41]. Hence the positive result of the ChIP experiment on this gene shows that Gfi-1 actually binds *in vivo *to Alu.

### Developmental and stress response TFs constitute a majority of the BSs on Alu

Most of the stress response TFs that bind to Alu are nuclear factors and hormone ligands that were validated experimentally, such as ER, RAR, LXRE and SP1 (Tables 2, 4). As was mentioned above, a subset of them are common to all Alu subfamilies while some appear only on the young Alu sequences, or on Alu elements that were mutated [20]. Another interesting group of TFs is developmental (usually referred to either as early markers or regulators of development); more than 30 members of this group passed the T5 threshold on at least one Alu consensuses sequence. The largest set of developmental TF binding sites that appear on Alu belong to hemateopoietic transcription factors [43] such as Gfi-1, LYF-1, GATA3, GATA2 and ONECUT2 (Table 4). Another set of BMs belong to TFs that are related to muscle and heart development [44], such as PITX2, MEF2, NKX2.5 and LUN1 (Tables 2, 4), and some to brain development: PAX4, NRL, OTX2 (Tables 2, 4). Note that even though the PAX6.01 PSSM did pass our thresholds (and hence appears in Table 4), it has been demonstrated [45] that PAX6 actually binds to very specific Alu sequences, that constitute only 0.2% of the genome-wide Alu elements. The PSSM that was used in our analysis (taken from MatInspector) was constructed from BMs that were identified in different experiments and its dominant binding sequences do not coincide with those that [45] identified as the Alu mediated PAX6 BMs.

### **The distribution of transposable elements in the 5' upstream regions of human genes**

**Figure 1:**
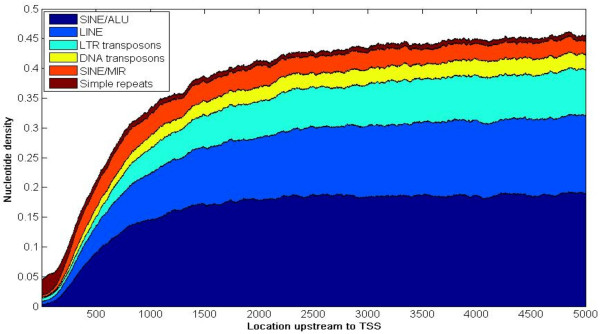
The nucleotide density of different repetitive elements in the 5 Kbp upstream to the TSS regions of 13686 genes. The average genomic Alu content is 10% and the nucleotide density associated with all the repetitive elements is about 45% [1].

The distribution of transposable elements in the 5' upstream regions of human genesis not uniform. We computed the density of different transposable elements on 5000 bp long upstream sequences of about 14000 genes. In the region between 2000 bp and 5000 bp from the transcription start site (TSS), 45% of the nucleotides belong to transposable elements. This fraction is the same as the overall density in the entire genome – see Fig 1. In contrast, in the region closer to the TSS, between 0 and 2000 bp – the density of transposable elements is lower. The Alu density exhibits a more interesting behaviour; for short distances – between 0 and 600 bp – it is lower than the genomic average of about 10%. This is not surprising since the immediate proximal region to the TSS is rich with important binding sites for initiating factors and insertion of Alu and other transposable elements in this region is most probably selected against. The density increases steadily till about 2000 bp, where it reaches a level of 18% of the base pairs [Fig. 1], after which it maintains a steady level (and has to decrease to the genomic average as we move further away into the intergenic region). The Alu content in the deep intergenic spacer regions is less than 10%; hence the density of on-Alu BSs in these regions is also lower than in the upstream 5000 bp regions of genes. The presences of less BSs in these regions might imply less functional relevance. Finally, 85% of the genes in our database contained at least one Alu insertion in the 5 Kbp upstream region.

### Non random distribution of Alu elements on promoter regions of different gene groups

We analyzed the distribution of Alu elements in the 5000 bp upstream regions of genes, treating separately groups of genes that belong to different biological processes. Protein biosynthesis genes are enriched with Alu compared to the genome-wide average value of 3.63 copies in the 5000 bp long upstream regions (Table 5). In contrast, genes that are related to various kinds of developmental processes, tissue formation (such as cell communication, central nervous system development genes and muscle development genes) contain a smaller number of Alu elements in their 5000 bp upstream region than the average value (see Table 5). Many of these differences between the Alu content of genes of a particular biological process and the Alu content of the entire genome (in the 5 Kbp upstream regions) were found (by t-test) to be highly significant (Table 5). As mentioned above, similar differences in Alu content were reported [12] when the full length gene sequences on chromosomes 21 and 22 were analyzed.

### Studying BS content in specific (non-consensus) Alu elements in the promoter regions

Hundreds of thousands of putative BSs reside on Alu elements in the upstream regions of genes. For several TFs one observes that nearly all the putative BSs (more than 90%) are located on Alu. The search for BMs presented in the previous sections was done on the consensus sequences of various Alu subfamilies. Since, however, Alu elements have mutated after insertion, there is considerable deviation from the consensus in the specific Alu sequences that are found across the genome. This variation may change the BS content of specific individual Alu elements versus the consensus. Hence we repeated our search of BSs in the actual 5000 bp upstream regions of about 14,000 genes, and if a BS passed a significance filter, we checked whether it resides on a specific Alu. This search was limited to those 66 BMs that passed our filter based on the consensus sequences, i.e. their largest score, Tmax, exceeded T5 for the consensus of at least one of the eight Alu subfamilies studied. For each of these 66 BMs we searched the 14,000 upstream regions for putative BMs whose score exceeds (the consensus-based) Tmax.

**Figure 2:**
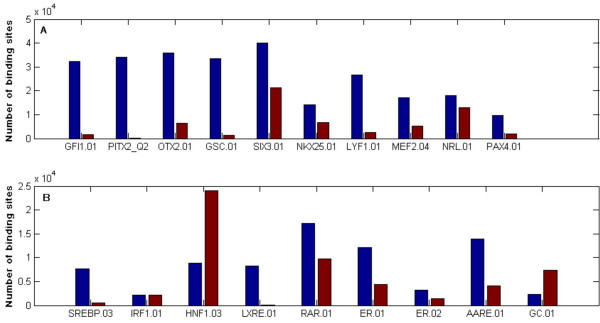
Number of BSs of 19 TFs (selected from the list of 66 – see text) on the 5 Kbp region upstream of 13686 genes. A DNA sequence was considered to be a BS if its score was higher than Tmax. The BS were counted separately for on- Alu (blue) and off-Alu (red) appearances. For 16 out of 19 TFs the majority of the putative BSs reside on Alu. (A) developmental TFs (B) other TFs.

We found that for 29 BMs out of the 66 the majority of putative BSs that pass Tmax reside on Alu sequences; the relative on and off Alu BM content is presented, for 19 out of these 66, in Fig 2. For example, for 5 out the 6 nuclear factors (Table 4) whose BMs appear among the 66, between 66% and 99% of the BMs (found in the 5000 bp upstream regions) are on Alu (see Fig. 2). The highest over-representations in Alu were observed for the BS of PITX2; over 99% of the 34000 BSs that pass the threshold were on Alu, and for LXRE's BSs over 99% of it's 9000 BSs that pass the threshold were on Alu, (see Fig. 2). In general, the distributions of putative BSs of TFs that bind to TGTAATCCC (Table 1) exhibit remarkable differences between the number of putative BS on and off Alu; over 90% of the total number of putative BSs of these TFs are located within Alu sequences. More moderate differences were found for developmental TFs such as SIX3.01, PAX4.01, NRL.01, MEF2.04 and NKX25.01 (Fig. 2). In general, for every BM that passed FDR of Q = 0.2 (see Table 1) over 80% of the BSs on the 5 kbp upstream region are located on Alu.

On the other hand, for the 37 (out of 66) remaining PSSMs the majority of BSs were not located on Alu, like IRF1.01 (48% out of the total number of 4000 BS were located on Alu) and HNF1.03 (27%). Still, even for these TFs, with mainly off-Alu BMs, the Alu repeats provide between hundreds to thousands of putative BSs in the 5 kbp upstream regions (see Table 6).

**Figure 3:**
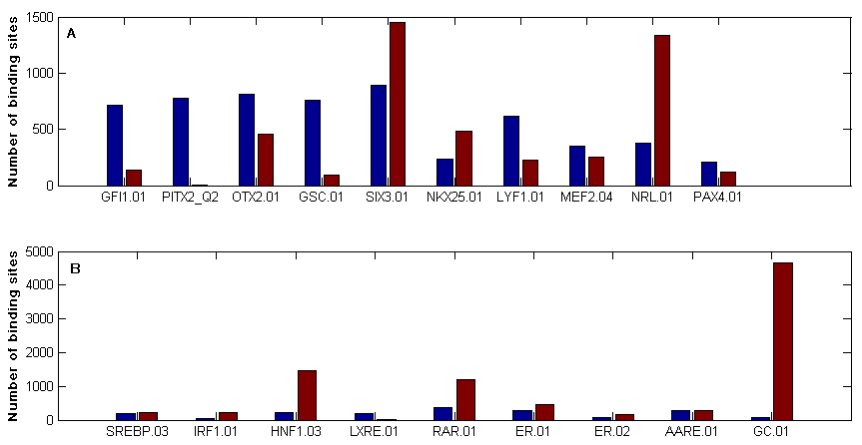
Number of BSs of 19 TFs (see text) on the 500 bp region upstream of 13686 genes. A DNA sequence was considered to be a BS if its score was higher than Tmax. The BS were counted separately for on (blue) and off Alu (red) 7 appearance. For 7 out of 19 TFs the majority of the putative BSs reside on Alu. (A) developmental TFs (B) other TFs.

As mentioned above, in the 500 bp upstream region, which constitutes the proximal promoter [46] and hence is believed to be most important for regulation, the Alu density is below the genome average (Fig. 1). To study this region in more detail, we repeated the analysis that was done for the 5000 bp region, and compared the distributions of BSs on and off Alu in the first 500 bp upstream to the TSS (see Fig 3 for 19 representative TFs). Only 9 out of the 66 BMs had more on Alu than off Alu BMs. These BMs include almost all homeodomain proteins, LXRE.01, LYF-1 and PAX4.01 (but not SIX3, see Fig. 3). In spite of the relatively low Alu density, on-Alu BMs do constitute a sizeable fraction of the putative BMs that are found in the 500 bp long upstream region (Fig. 3 and Table 6).

### Genes of different biological processes contain different amounts of BSs as a result of differences in their Alu content

**Figure 4:**
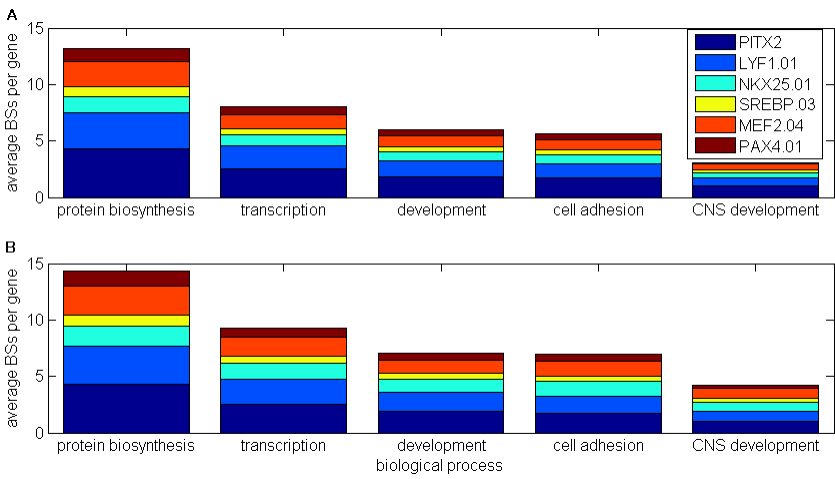
We present for 6 TFs the number of putative BSs in the 5 Kbp region upstream, averaged over the genes that belong to various biological processes. The 6 TFs shown were chosen because their BSs are non-overlapping and their Tmax is greater than T5 (see text) a. for on-Alu BSs b. all BSs(on and off Alu).

We used the results of the search for on and off Alu BMs to look for differences between genes that belong to different biological processes. As expected, for those biological processes whose genes were previously found to be enriched with Alu (Table 6), we also found that a high percentage of their regulatory signals were on-Alu. The Alu rich groups in this category include metabolic and bio-synthesis associated genes. Since for several TFs a large majority of BMs reside on Alu, we find that Alu-rich functional families have a large number of these Alu-associated BMs, whereas Alu-poor biological processes (such as developmental genes) will have less of these BMs (see Fig. 4 and Additional data file 2). For example, the average number of putative BSs of PITX2 is almost 4 times greater in protein biosynthetic genes than in CNS developmental genes (Fig. 4). Interestingly, as a result, developmental TFs such as LYF-1, PAX4, MEF2D and PITX2, that have on-Alu BMs, appear less in the (Alu-poor) upstream region of developmental genes than in the Alu-rich non-developmental group of protein biosynthesis related genes. One possible explanation of this may be that the developmental TFs main role is to suppress pathways that are in competition with or exclusive of the developmental process (versus acting directly on development – associated genes).

One should emphasize that the statements made above are based mainly on in-silico evidence, which must be supplemented by firm experimental observations of binding and functional roles of the bound TFs.

### Inter-species comparisons: B1, the murine 'Alu like' elements, contain several BSs that are similar to the primate Alu BSs

We scanned the B1 consensus sequences that were downloaded from RepBase [29]. These sequences were less enriched with BSs that pass the T5 threshold; this might be due to the fact that the length of the B1 sequences is only half that of Alu. Twenty three binding motifs passed T5 on B1 (see Table 7) and we found that 11 of these passed T5 also in Alu, as expected from the similarity between B1 and Alu [47] (bold in Table 7). One of these shared BSs was T(G/T)TAATCCC where G appears in all Alu families and T appears in the consensus of B1s. This BS is the target of OTX2.01, a homeodomain protein in both human and mouse. An additional homeodomain protein, CRX01.01, is also bound to this BS in mouse; it did not pass T5 in Alu. The members of the MEF2 family of BMs appear in mouse (see Table 7) with a weaker score than in Alu, (unpublished data). The PSSM with the most significant score on B1 was PAX6.02, which is not surprising since this PSSM is built from those BSs of PAX6 that were known to reside on B1 [48]. In addition, a group of BMs that are related to nuclear factors and response proteins passed T5 on B1 (TTF1.01 and ERR_01) but not on Alu. This is surprising since there are experiments that validated that the TFs that correspond to these BMs bind to primate Alu [16].

### The distributions of B1 in mouse promoters and Alu in human promoters are positively correlated

**Figure 5:**
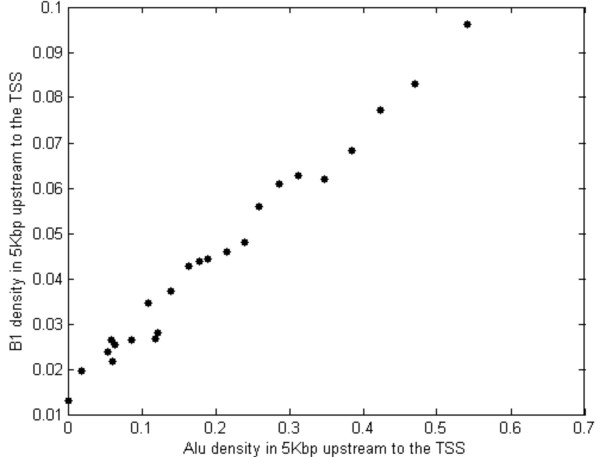
Scatter plot of nucleotide densities in 5 Kbp upstream of the TSS, that belong to B1 (in mouse genes) and to Alu (in orthologous human genes). For both species the values presented by a point are obtained by averaging over 200 genes that belong to a "gene set". There are 27 gene sets, that were obtained by sorting the 5400 human promoters (that have mouse othologues) according to their Alu content, and dividing them into 27 bins (of 200 genes in each). Each mouse gene set contains the orthologous genes of the corresponding human gene-set.

When the mouse genome was first sequenced, regions orthologous to human DNA were identified. The densities of B1 and B2 in mouse and Alu in human were measured in windows of 500,000 bp and the Alu density in humans was found to be correlated with B2 and B1 density in mouse [49]. A high correlation was quite unexpected, since Alu and the mouse SINE elements B1 and B2 spread in the rodent and human genomes *after *the divergence of rodents and primates. We checked whether this trend is present also in the regulatory regions of genes, of size 5 kbp, and we found a positive correlation between B1 and ALU densities even in this relatively short region. In more than 5400 pairs of orthologous genes, that had available upstream sequences in both human and mouse, we found positive correlation (see methods) of 0.495 (pval<1e-05). In addition we ordered the genes by increasing Alu content in the 5000 upstream bp region, and divided the genes on this basis into 27 sets, with each set containing 200 genes. We then computed the average Alu and B1 associated nucleotide densities for each of the 27 sets, and plotted the two average Alu/B1 contents (Fig 5). Each point in Fig 5 represents a group of 200 genes, with the x (y) coordinates representing the Alu (B1) densities in human (mouse). The monotonic behaviour evident in Fig 5 indicates that the Alu and B1 contents of orthologous genes are largely similar in human and mouse. This similarity is far from being obvious, since spreading of Alu and B1 across the genome is believed to have happened *after *the divergence of primates and rodents.

## Conclusion

### Summary

This research suggests that evolution used transposable elements to insert modules of transcription factor binding motifs into promoters and, by means of their presence, assemble higher level regulatory networks. In order to explore this question we focused on Alu elements, which are good potential candidates to be part of the building blocks of regulatory networks for two reasons. First, Alu elements are abundant in the upstream region of the TSS of genes, and second, Alu elements contain dozens of putative BSs for TFs. Some of these BSs were found before and their association with Alu was also reported, whereas in some cases although the BSs were found, the fact that they reside on Alu went unnoticed. Finally, we list here also BSs on Alu that were not identified previously. Our findings imply that the biological pathway on which Alu-mediated regulation appears to have the most significant impact is the development process. Many of the TFs that have binding motifs on Alu are associated with development; moreover, some of these BSs were previously demonstrated to be functional *in vivo *and essential to regulation of some target genes.

We have also shown that a few subsequences on Alu, that were known to be highly conserved, provide many of the binding sites for the transcription factors. We studied the Alu distribution in the upstream region of genes and found that while Alu are "repelled" from the 500 bp long region immediately preceding the transcription starts site, the 4500 bp long sequence further upstream is enriched (compared to the entire genome). The Alu content in the promoters varies between functional families of genes.

Comparison of Alu in human and B1 in mice shows a significant correlation between their distributions (with respect to orthologs), and high similarity between the binding motifs that reside on the two mobile elements.

Several particular cases (TFs and genes) were discussed in detail.

### Future directions

Most importantly, more experiments are needed to verify that the BMs that were found by us (see Table 1 and Additional data file 1) indeed bind their corresponding TFs and, furthermore, to verify their regulatory roles. Of particular interest is the role of Alu in developmental processes. We found that promoters of genes involved in biosynthesis and metabolism are enriched (via Alu) in BMs of several developmental TFs (Table 4). It is our belief that since development and cell proliferation (of which biosynthesis is an essential component) are opposing processes [50], these BMs play an inhibitory role, suppressing proliferation during differentiation. This hypothesis is supported by the known suppressory role of GFI1, but the presently known activities on PITX2 and NKX2.5 do not fit this picture. Further experiments that address this issue by measuring the regulatory effects of other Alu-associated developmental TFs are needed. Clearly, many of the genes and their functions date earlier than the rodent/primate divergence, whereas the Alu insertions happened later. This poses an open and debated [20,51] question about the mechanism by which pre-existing regulatory circuits must have been replaced, modified or supplemented by Alu insertions.

## Methods

### Downloads

The Alu consensus sequences were downloaded from Repbase [29].

To scan for BMs we downloaded 410 vertebrate PSSMs from the MathInspector [30] database, with two additional PSSMs ($PITX2_Q2 and &LUN1_01) taken from the TRANSFAC database [31].

The coordinates of the upstream regions of 21000 human genes were taken from [52]. The annotations of the U133 Array (Affymetrix, Santa Clara, CA) were used in order to remove multiple upstream regions for different splice variants of the same gene, (a single upstream region was associated with each gene symbol). We also removed from our database those Refseq genes that have duplicates on chromosomes X and Y, and those with upstream regions that were not fully sequenced. All this left 13686 promoters of different Refseq genes for analysis.

The coordinates of Alu elements and other transposable elements in the human genome were extracted from the file chromOut.zip, which was downloaded from [52]. This is the output file of RepeatMasker [53]. The sequences of the Alu that reside in promoters were then extracted from the human genome, by intersecting these Alu coordinates with the upstream sequences of our 13686 genes.

Similarly, for mouse we downloaded from [54] the files upstream5000.zip which contained the coordinates and sequences of the regions upstream to the TSS of mouse genes, and the RepeatMasker output file of the mouse genome, chromOut.zip. We used annotations of Mouse 430_2 Array (Affymetrix, Santa Clara, CA) in order to remove multiple promoters.

In order to annotate genes to biological processes and molecular functions we used the DAVID database (based on the GO annotation) [55].

### Calculating scores and probabilities

For each PSSM we calculated the maximal score, on both sense and antisense orientations.

In order to find the probability of getting some maximal score for a BM on an Alu sequence we produced 100,000 random 281 bp long sequences, of di-nucleotide distribution similar to that of the AluSx consensus sequence. The next step was to calculate the maximal score for a given PSSM for each random sequence. The p-value for each given score was the percent of random sequences that contained a target site with higher score. Although, such procedure might seem to be biased towards AluSx, it can be seen in Tables 8 and 9 that the subfamilies on which we focus in this research have similar nucleotide and di- nucleotide contents. Note that since we did not use a twice repeated random sequence of 140 nucleotides, our p-values are an upper bound to the true ones.

### Orthologous genes in mouse and human

We compared gene symbols of human and mouse and got about 5400 orthologous gene pairs and their respective promoters in human and mouse. The Pearson's correlation coffeeicient between the Alu and B1 densities on human and mouse genes was calculated over these 5400 pairs of promoters.

## Authors' contributions

PP participated in the conception of the study, carried out the mapping of the Alu elements and BSs in the upstream of genes, analyzed the data, wrote the initial draft of the manuscript. ED conceived of the study, and participated in its design coordination and supervision, helped to draft and edited the manuscript.

## Supplementary Material

Additional File 1 Sixty six binding motifs whose scores pass T5 on at least one major Alu subfamily sequence. The consensus sites of the PSSMs (from the IUPAC convention [33]) is in the second column and the the target sequences with the highest scores among all the major Alu consensus sequences – in the third, with nucleotides that agree with the consensus denoted by capital letters. The fourth column contains the locations of the putative target sites on the Alu sequences of the subfamily with the highest score (in case the same sequence appears on several Alu elements, we choose the one with the highest number of copies in the 5 kb upstream regions). Two Alu subsequences serve as putative target sites of several TFs (designated by *, and **). The fifth column contains the p-values (see text and methods) and the number of subfamilies on which the BSs of the third column resides is listed in column 6. Click here for file

Additional File 2 We present for 66 PSSMs the number of putative BSs in the 5 Kbp region upstream, averaged over the genes that belong to various biological processes. The PSSMs that are shown are those for which Tmax>T5. The number of genes in each biological process is given in column 2. The Alu density and number of Alu repeats per gene are given in columns 3 and 4 respectively. Click here for file

## Figures and Tables

**Table 1:** Twenty five binding motifs whose scores pass T1. Those that pass FDR of Q = 0.20 on at least one major Alu subfamily sequence are in bold. The consensus sites of the PSSMs (from the IUPAC convention [33]) is in the second column and the target sequences with the highest scores among all the major Alu consensus sequences – in the third, with nucleotides that agree with the consensus denoted by capital letters. The fourth column contains the locations of the putative target sites on the Alu sequences of the subfamily with the highest score (in case the same sequence appears on several Alu elements, we choose the one with the highest number of copies in the 5 kb upstream regions). Two Alu subsequences serve as putative target sites of several TFs (designated by*, and **). The fifth column contains the p-values (see text and methods) and the number of subfamilies on which the BSs of the third column resides is listed in column 6.

**BM name**	**Consensus**	**Binding site**	**location**	**p-val**	**n. fam**
**V$OTX2.01**	NNGGATTAANNN	TGGGATTAcAGG	AluSx (-22:-33)*	4.00E-03	8
**V$GSC.01**	ANGRGATTASMN	cTGGGATTACAG	AluSx (-23:-34)*	2.60E-03	8
**V$GFI1.01**	NNAAATCNNAGNN	TGtAATCCCAGCA	AluSx (24:36)*	2.00E-03	8
**V$PITX2_Q2**	WNTAATCCCAR	TGTAATCCCAG	AluSx (24:34)*	1.00E-05	8
**V$LUN1_01**	TCCCAGCTACTTTGGGA	TCCCAGCactTTgGGa	AluSx (29:45)	1.00E-05	8
**V$LYF1.01**	TTTGGGARR	TTTGGGAGG	AluSx (38:46)	1.70E-03	8
**V$LXRE.01**	NGNTYACTNNMGKTCA	GGATCACcTGAGGTCA	AluSx (58:73)	1.00E-04	2
**V$SREBP.03**	NATCACGTGAN	GATCACcTGAG	AluSx (59:69)	1.60E-03	3
V$RORA1.01	NNWWNNAGGTCAN	ATcACGAGGTCAA	AluSc (60:72)	8.50E-03	1
**V$NKX25.01**	TYAAGTG	TCAAGTG	AluJb (-62:-68)	1.00E-05	1
V$HNF4.02	NNGGNCNAAAGNTCN	GAGGTCAAgAGATCG	AluSc (65:79)	6.60E-03	1
**V$ER.02**	NNAGGTCANNGTGACCT	GTtGGTCAGGcTGgtCT	AluSp (-82:-98)	4.90E-03	1
V$CHREBP _MLX_01	CAYGNGNNANNSNNGTG	CACGGtGAAACCCCGTc	AluY (96:112)	6.50E-03	1
**V$AARE.01**	RTTKCATCA	GTTTCAcCA	AluSx (-100:-108)	3.60E-03	5
**V$MEF2.04**	TGTTACTAAAAATAGAAM	ctcTACTAAAAATAcAAA	AluSx (114:131)**	4.00E-04	6
**V$MEF2.03**	NNKWKCTAWAAATAGMNN	CTcTaCTAAAAATAcAAA	AluSx (114:131)**	3.50E-03	6
**V$MEF2.02**	NNNCTAWAAATAGMNN	CTACTAAAAATAcAAA	AluSx (116:131)**	2.60E-03	6
**V$RSRFC4.01**	NWKCTAWAAATAGMNN	CTaCTAAAAATAcAAA	AluSx (116:131)**	3.30E-03	6
**V$RSRFC4.02**	ANKCTAWAAATAGMWNN	cTaCTAAAAATAcAAAA	AluSx (116:132)**	3.00E-03	6
**V$OC2.01**	NNANANAATCMANANNT	ACAAAAAATaCAAAAAT	AluJo (118:134)**	4.00E-04	2
V$BRN2.03	NNMTWNATTWNMWTN	ACAaAAATTAGCTgG	AluSc (125:139)**	8.30E-03	1
V$HNF1.01	GGTTAATNWTTAMM	GGcTAATTTTTgtA	AluSx (-126:-139)**	8.80E-03	6
V$HNF1.03	GGTTAATNWTTRNC	GGcTAATTTTTGTa	AluSx (-126:-139)**	6.60E-03	6
**V$PAX4.01**	NAMWAATTASS	AAAAAATTAGC	AluY (127:137)**	4.00E-04	1
**V$IRF1.01**	SNAAAGYGAAACY	CAAgAGCGAAACT	AluSp (264:276)	3.60E-03	2

**Table 2:** TFs for which the binding to the Alu-associated BM has been experimentally verified. Most passed T1 and FDR of 20%. The last two (marked by **) did not pass our promoter regions search, but were reported in the literature as bound to Alu. Four TFs (**bold**) are new: they were found by us but their being bound to Alu went unnoticed. The first column is the PSSM symbol from MatInspector [30] or TRANSCFAC [31] the second and third columns are the gene symbol and description of the biological process. The last two columns list those target genes of each TF that have on-Alu BSs, and the appropriate references.

**Binding Motif name**	**TF Gene Symbol**	**Biological process involvement**	**Target genes with Alu target sites**	**Ref**
**V$PITX2_Q2**	**PITX2**	Eye, heart, tooth and abdominal organs	*PLOD1*	[40,42]
**V$NKX25.01**	**Nkx2.5**	Heart formation	*PLOD1*	[42]
V$LYF1.01*	ZNFN1A1 (LYF1)	T-cell differentiation	*CD8*	[27]
**V$GFI1.01**	**Gfi-1**	Hematopoietic differentiation	*AZU, P21, JAK3, ACT, AAT*	[41]
V$LXRE.01*	*LXREα*			[56]
V$ER.01–02	*ER*		*BRCA1, ERF-3*	[18]
V$RAR.01*	*RARA*	Retonic acid receptors family. Embryonic morphogenesis	*Keratin K18*	[22]
V$GC.01*	*SP1*	Nuclear factor. Tumer suppressor		[21]
**V$LUN1**	***LUN1***	Lung development		[39]
V$PAX6.02**	PAX6**	Pancreas development		[45]
V$YY1.01**	YY1**	Nuclear factor		[20]

**Table 3:** List of human target genes of (murine) Gfi-1 which contain Alu in their 5' upstream region. In these genes some of the Gfi-1 target sites are on Alu sequences; such sequences are denoted by '+' in the third column. The first column contains the target gene name; the second column is a potential binding sequence in the promoter region of the gene. The fourth column is a result of EMSA binding experiment for the sequence in column 2 [41].

**Gene**	**Potential Gfi-1 BSs**	**part of Alu**	**Binding in EMSA**
AZU	1, 5'-CTCCCAAAGTG**CTGAGATTA**CAGGTGTGAG-3'^†^	+	+
	2, 5'-CGGCCTGGAG**ACCAGATTT**GAGCCCTGGGA-3'	-	+
	3, 5'-ACTTCCTGCCTC**TGCAGATTT**GCTTGTTCT-3'	-	-
	4, 5'-TTTCACACCTG**TAATCCCAG**CATTTTGGGA-3'	+	+
	5, 5'-GGTGGACAACTG**TAATCCTAG**CTACTCAGG-3"	-	-
AAT	1, 5'-GCACACGCCTG**TAATCCCAG**CTATTTGGGA-3'	+	+
	2, 5'-CCCAGGAGGTT**TAATCACAG**CCCCTCCATG-3'*	-	+
	3, 5'-ATAGCTGACC**TAATCTCTC**TGGCTTTGGTT-3'*	-	-
ACT	1, 5'-GCTCATGCCTG**TAATCCCAG**CACTTTGGGA-3'	+	+
	2, 5'-TTCTTGGCTGCCACTGATTCCCTGTGCCTT-3'	-	+
	3, 5'-GCCAAAATA**AAATCCCAG**TCCAAGTCTGGG-3'*	-	+
Jak3	1, 5'-CTCACGTGTG**TAATCACAG**CACTTTGGG-3'	+	+
P21	1, 5'-GCATGCCTG**TAATCCCAG**CTACTCGGG-3'	+	+
	2, 5'-ATGTCTGGG**CAGAGATTT**CCAGACTCT-3'	-	+

*These sites were tested in transient transfection assays.

**Table 4:** List of biological processes whose regulation involves transcription factors with putative target sites on Alu. Those transcription factors that were experimentally found to bind to Alu (see Table 2) are in **bold**. These reported binding events involved specific Alu sequences, and were not based on genome-wide screening.

**Biological Porcess**	**BM(*TFs*)**
Nuclear factors and stress response	**ER.01–2 **(see table 2), **RAR.01 **(table 2), **LXRE.01**, GC.01 (*SP1*)
Hematopoietic differentiation	GFI.01 (Gfi-1*) LYF1.01 (*ZNF1A1*), GATA3.02 (*GATA3*), OC2.01 (*ONECUT2*), GATA2.01(*GATA2*)
Heart development/Muscle development	PITX2*, NKX25.01 (Nkx2.5*), LUN01_01*(LUN1) *MEF2.01–04 (*MEF2A,B,C,D*), RSRFC4.01/02
Brain and CNS development:	**PAX6.01 (PAX6)**,PAX4.01 (*PAX4*), OTX2.02 (*OTX2*), NRL.01 (NRL), BRN2.03, (*POU3F2*), BRN3.01(POU3F3)
Eye development	PITX2.Q2 (*PITX2*)*,PAX6.01 (*PAX6*), SIX3.01(*SIX3*)
Pancreas developments	**PAX6.01(*PAX6*)**, PDX1.01
Embryonic development	GSC.01 (*GSCL*)
Sterol biosynthesis	SREBP.03 (*SREBPF3*)
Interferon	IRF1.01 (*IRF1) *IRF2.01*,(IRF2*), HEN1.01, HEN1.03 (*TCF1,TCF2*)

**Table 5:** List of biological processes and statistics of the corresponding Alu content in the 5 kbp upstream regions of the associated genes.

**Biological process**	**number of genes**	**number of Alu**	**Average number per gene**	**Ttest p-value**
central nervous system development	86	167	1.94	1.73E-06
skeletal development	105	210	2.00	2.13E-07
potassium ion transport	128	270	2.11	9.41E-08
organogenesis	816	1986	2.43	4.63E-25
cell-cell adhesion	114	281	2.46	1.28E-04
neurogenesis	335	835	2.49	2.20E-10
cell-cell signaling	470	1190	2.53	3.18E-13
morphogenesis	1010	2606	2.58	9.42E-24
cell adhesion	471	1288	2.74	2.54E-09
muscle development	150	412	2.74	7.95E-04
sensory perception	234	646	2.76	3.76E-05
ion transport	573	1595	2.78	5.06E-10
inflammatory response	181	507	2.80	5.50E-04
development	1519	4274	2.81	3.92E-21
immune response	635	1853	2.92	4.21E-08
response to external stimulus	910	2727	3.00	7.05E-09
cell communication	2535	7885	3.11	3.66E-14
signal transduction	2017	6538	3.24	2.79E-07
genome	13686	49748	3.63	1
metabolism	5605	21936	3.91	6.85E-08
cell cycle	651	2751	4.23	5.56E-06
biosynthesis	899	3827	4.26	2.92E-08
intracellular transport	406	1840	4.53	3.86E-08
protein localization	418	1981	4.74	7.15E-12
protein biosynthesis	452	2155	4.77	3.24E-13
mrna processing	205	1006	4.91	2.37E-08
rna processing	322	1611	5.00	6.70E-14
rna splicing	173	872	5.04	1.42E-08
rna metabolism	391	1987	5.08	3.66E-18
ribosome biogenesis	43	223	5.19	1.62E-03
translation	138	743	5.39	2.62E-10

**Table 6:** Amount of binding motifs (for 25 TFs with Tmax > T1) in the 500 bp and 5000 bp regions of 13686 genes. For each BM we show statistics with respect to sequences that passed Tmax and T5 (see text for further details). We also checked whether the BS resides on Alu or not (see methods). and give the number of BSs that reside on Alu and their percentage out of the total number of BS.

	5000 upstream				500 upstream			
BM name	No. of BS on Alu – Tmax	%BS on Alu- Tmax	No. of BS on Alu T5	%BS on Alu T5	No. of BS on Alu Tmax	%BS on Alu Tmax	No. of BS on Alu T5	%BS on Alu T5

V$PITX2_Q2	34188	99.4%	45546	94.9%	777	99.0%	1022	86.0%
V$LXRE.01	8193	99.2%	21686	74.9%	185	96.9%	473	42.2%
V$GSC.01	33565	96.0%	36033	90.7%	761	89.2%	812	75.5%
V$GFI1.01	32311	95.2%	33557	88.3%	713	84.0%	742	68.5%
V$SREBP.03	7695	93.0%	18264	65.9%	181	44.7%	399	18.8%
V$LYF1.01	26778	91.8%	27476	84.0%	616	73.5%	624	56.1%
V$OTX2.01	35993	84.8%	35993	84.8%	813	64.0%	813	64.0%
V$PAX4.01	9760	84.0%	23265	74.7%	207	63.9%	508	53.4%
V$AARE.01	13854	77.3%	17047	62.0%	282	51.1%	328	30.5%
V$MEF2.04	17253	76.7%	19766	65.2%	355	58.7%	414	43.6%
V$CHREBP_MLX_01	1537	70.3%	9810	68.6%	49	23.6%	261	20.5%
V$ER.02	3166	68.2%	12256	50.7%	60	27.6%	260	15.9%
V$NKX25.01	14144	67.7%	14754	48.9%	238	32.9%	249	18.7%
V$LUN1_01	15768	99.9%	50303	99.6%	340	100.0%	1143	98.1%
V$HNF4.02	2224	61.5%	11368	61.0%	39	18.9%	259	21.6%
V$MEF2.02	18807	56.9%	18807	56.9%	389	30.7%	389	30.7%
V$RSRFC4.01	18153	49.5%	18153	49.5%	381	26.3%	381	26.3%
V$RSRFC4.02	18424	49.0%	18424	49.0%	385	26.7%	385	26.7%
V$IRF1.01	2116	48.8%	5343	19.6%	57	20.0%	144	7.1%
V$MEF2.03	18027	48.2%	18027	48.2%	383	26.3%	383	26.3%
V$HNF1.03	8819	26.9%	8819	26.9%	216	12.9%	216	12.9%
V$OC2.01	4430	25.5%	8643	27.1%	98	15.2%	204	16.6%
V$RORA1.01	968	12.8%	6891	26.7%	25	5.8%	152	10.6%
V$HNF1.01	2939	10.3%	2939	10.3%	74	4.5%	74	4.5%
V$BRN2.03	2127	6.8%	2127	6.8%	60	4.0%	60	4.0%

**Table 7:** BMs with target sites on B1_mM and B1_Mus ('Alu Like' sequence in mouse). The scores of the target sites were above T5 (those for which the corresponding ALU BS had scores greater than T5 are in bold). The IUPAC convention [33] consensus sites are in column 2, and the third column shows the target site with the highest score among all the major consensus sequence (nucleotides that agree with the consensus are denoted with capital letters). The location of the putative target sites is in column 4, given for the subfamily on which the target site of column 3 appears. Column 6 lists the number, n, of B1 subfamilies on which the BS shown in column 3 is located.

BM name	Consensus	Binding site	location	p-val	n
**V$ATF6.01**	SNGCCACNNNNNN	CCaCCACGCCCGG	B1_Mm (-3:-15)	2.8E-02	1
V$ZBRK1_01	SGGGSMRCAGNYMTTTKTKKSC	GGtGGCGCAcGCCTTTaaTcCC	B1_Mm (11:32)	2.9E-02	2
**V$BRN3.02**	NNYYWKTAATYWNWY	CGCCTTTAATCcCAg	B1_Mm (20:34)	3.9E-02	2
V$GKLF_02	NTYAMAGGRN	ATTAAAGGcG	B1_Mm (-20:-29)	1.1E-02	2
V$CRX.01	NNNGATTARNNT	TGGGATTAAAGg	B1_Mm (-22:-33)	7.9E-03	2
**V$OTX2.01**	NNGGATTAANNN	TGGGATTAAAGG	B1_Mm (-22:-33)	3.0E-04	2
V$TTF1.01	NNWSTCAAGYRYWN	CCTCcCAAGTGCTG	B1_Mus1 (-33:-46)	4.6E-03	1
V$LEF1.02	NNNWTCAAAGN	GTCTaCAAAGT	B1_Mm (83:93)	1.4E-02	1
V$PAX6.02	NNAGKKCCAGGNNMG	TGAGTTCCAGGACAG	B1_Mm (93:107)	1.0E-05	2
**V$ERR_01**	NNTCAAGGTCASNN	GTTCcAGGaCAGCC	B1_Mm (96:109)	3.9E-02	2
V$GRE.01	NNGGTWCNNNNTGTTCTNR	GTaGccCTGGCTGTcCTGG	B1_Mus1 (-100:-118)	1.9E-02	1
V$CP2.02	NWCYGSNNMWNNCTNGNY	CTCTGtATAgCCCTGGCT	B1_Mm (-106:-123)	1.7E-02	1
V$MTATA.01	NNNTWTAAANCNNNNNN	CTCTgTAtAGCCCTGGC	B1_Mm (-107:-123)	4.6E-02	1
**V$MEF2.04**	NNTGTTACTAAAAATAGAAMNN	AGgGTTtCTctgtATAGccCTG	B1_Mm (-109:-130)	3.4E-02	1
**V$RSRFC4.02**	ANKCTAWAAATAGMWNN	tTTCTcTgtATAGCcCT	B1_Mm (-110:-126)	4.9E-02	1
**V$MEF2.03**	NNKWKCTAWAAATAGMNN	GGTTTCTcTgtATAGCCC	B1_Mm (-111:-128)	3.9E-02	1
**V$RSRFC4.01**	NWKCTAWAAATAGMNN	TTTCTcTgtATAGCCC	B1_Mm (-111:-126)	4.9E-02	1
**V$AMEF2.01**	NNNNNTAAAAATANCNNN	CTGTCTcgAAAaAACAAA	B1_Mm (129:146)	1.1E-02	2
V$FAST1.01	NNTTGTKKATTGGS	TTTTGTTTtTTcGa	B1_Mm (-134:-147)	3.9E-02	2
V$HFH8.01	YRNATAAACANN	CGAAaAAACAAA	B1_Mm (135:146)	1.1E-02	2
**V$XFD2.01**	NNWATAAACANNN	CGAAaAAACAAAA	B1_Mm (135:147)	4.5E-02	2
V$HFH1.01	AWATAAACAWTN	gAAaAAACAAaA	B1_Mm (136:147)	2.7E-02	2
**V$HFH2.01**	RAANAAAYAWTN	GAAAAAACAAaA	B1_Mm (136:147)	2.0E-03	2

**Table 8:** The nucleotides distribution in the consensus sequences of major Alu subfamilies.

ALU name	ALU length	C	G	T	G + C
AluJo	283	28.62%	33.92%	16.25%	62.54%
AluJb	283	28.98%	34.63%	15.55%	63.60%
AluSc	280	28.57%	33.57%	15.71%	62.14%
AluSg	281	28.83%	34.16%	15.30%	62.99%
AluSp	284	28.17%	33.10%	15.85%	61.27%
AluSq	284	28.17%	33.45%	15.85%	61.62%
AluSx	283	28.98%	33.92%	15.55%	62.90%
AluY	282	28.37%	34.75%	14.89%	63.12%

**Table 9:** Number of di -nucleotide appearances in the consensus sequences of major Alu subfamilies.

ALU NAME	ALU length	AA	AC	AG	AT	CA	CC	CG	CT	GA	GC	GG	GT	TA	TC	TG	TT
AluJo	283	11	12	28	8	18	23	21	19	23	33	29	11	8	13	17	8
AluJb	283	13	13	25	7	18	24	22	18	22	31	32	13	6	14	18	6
AluSc	280	15	14	23	9	17	21	24	18	23	29	30	12	7	16	16	5
AluSg	281	15	14	23	8	17	22	25	17	22	30	32	12	7	15	15	6
AluSp	284	19	14	21	10	17	23	23	17	22	28	33	11	7	15	16	7
AluSq	284	19	14	22	8	18	23	21	18	20	28	34	13	7	15	17	6
AluSx	283	15	14	23	8	17	23	24	18	22	30	32	12	7	15	16	6
AluY	282	15	15	23	8	16	21	25	18	23	29	34	12	8	15	15	4
